# Emerging role of IGF1R and IR expression and localisation in adrenocortical carcinomas

**DOI:** 10.1186/s12964-025-02115-0

**Published:** 2025-03-04

**Authors:** Rosa Catalano, Emma Nozza, Barbara Altieri, Emanuela Esposito, Giorgio A. Croci, Anna Maria Barbieri, Donatella Treppiedi, Sonia Di Bari, Otilia Kimpel, Mario Detomas, Mariangela Tamburello, Marc P. Schauer, Sabine Herterich, Anna Angelousi, Michaela Luconi, Letizia Canu, Gabriella Nesi, Constanze Hantel, Sandra Sigala, Laura-Sophie Landwehr, Guido Di Dalmazi, Elisa Cassinotti, Ludovica Baldari, Serena Palmieri, Alessandra Mangone, Emanuele Ferrante, Cristina L. Ronchi, Giovanna Mantovani, Erika Peverelli

**Affiliations:** 1 Department of Clinical Sciences and Community Health, University of Milan, 20122 Milan, Italy; 2 PhD Program in Experimental Medicine, University of Milan, 20122 Milan, Italy; 3 Division of Endocrinology and Diabetes, Department of Internal Medicine I, University Hospital, University of Würzburg, 97080 Würzburg, Germany; 4 Pathology Unit, Fondazione IRCCS Ca’ Granda Ospedale Maggiore Policlinico, 20122 Milan, Italy; 5 Endocrinology Unit, Fondazione IRCCS Ca’ Granda Ospedale Maggiore Policlinico, 20122 Milan, Italy; 6 Section of Pharmacology, Department of Molecular and Translational Medicine, University of Brescia, 25121 Brescia, Italy; 7 Central Laboratory, University Hospital of Würzburg, 97080 Würzburg, Germany; 8 First Department of Internal Medicine, Laikon General Hospital, Medical School, National and Kapodistrian University of Athens, 11527 Athens, Greece; 9 51st Department of Propaedeutic Internal Medicine, National Technical University of Athens, Mikras Asias 75, Athens, 11527 Greece; 10 Endocrinology Unit, Department of Experimental and Clinical Biomedical Sciences “Mario Serio”, University of Florence, 50139 Florence, Italy; 11 Centro di Ricerca e Innovazione sulle Patologie Surrenaliche, Azienda Ospedaliero-Universitaria Careggi, 50134 Florence, Italy; 12 Department of Endocrinology, Diabetology and Clinical Nutrition, University Hospital Zurich (USZ) and University of Zurich (UZH), Zurich, CH-8006 Switzerland; 13 Medizinische Klinik und Poliklinik III, University Hospital Carl Gustav Carus Dresden, 01307 Dresden, Germany; 14 Division of Endocrinology and Diabetes Prevention and Care, IRCCS Azienda Ospedaliero-Universitaria di Bologna, 40138 Bologna, Italy; 15 Department of Medical and Surgical Sciences (DIMEC), Alma Mater Studiorum University of Bologna, 40126 Bologna, Italy; 16 Department of Surgery, Fondazione IRCCS Ca’ Granda Ospedale Maggiore Policlinico, 20122 Milan, Italy; 17 Department of Metabolism and System Science, University of Birmingham, Birmingham, B15 2TT UK; 18 Centre for Endocrinology, Diabetes and Metabolism (CEDAM), Birmingham Health Partners, Birmingham, B15 2TT UK

**Keywords:** Adrenocortical carcinoma, IGF1R, Insulin receptor, Biomarker, Cellular localisation

## Abstract

**Background:**

The insulin-like growth factor 2 (IGF2) is overexpressed in 90% of adrenocortical carcinomas (ACC) and promotes cell proliferation via IGF1R and isoform A of insulin receptor (IRA). However, IGF2 role in ACC tumourigenesis has not been completely understood yet, and the contribution of IGF1R and IRA in mediating ACC cell growth has been poorly explored. This study aimed to investigate IGF1R and IR expression and localisation, including the expression of IR isoforms, in ACC and adrenocortical adenomas (ACA), and their role in IGF2-driven proliferation.

**Methods:**

Immunohistochemistry staining of IGF1R and IR was performed on 118 ACC and 22 ACA to evaluate their expression and cellular localisation and statistical analyses were carried out to assess correlations with clinicopathological data. The expression of IRA and IRB in ACC and ACA tissues, ACC cell lines and ACC and ACA primary cultures was determined by RT-qPCR. To appraise the specific role of IGF1R and IR in mediating IGF2 mitogenic pathway, single and double silencing of receptors and their inhibition in 2 ACC cell lines derived from primary tumours (H295R and JIL-2266) and 2 derived from metastatic tumours (MUC-1 and TVBF-7) as well as in ACC and ACA primary cultures were performed.

**Results:**

We found a higher IGF1R plasma membrane localisation in ACC compared to ACA. In ACC this localisation was associated with higher Ki67 and Weiss score. IR was expressed in about half of ACC and in all ACA but, in ACC, it was associated with higher Ki67 and Weiss score. RT-qPCR revealed that the prevalent isoform of IR was IRA in ACC and ACA, but not in normal adrenals. In ACC cell lines, double IGF1R + IR silencing reduced cell proliferation in JIL-2266, MUC-1 and TVBF-7 but not in H295R. In ACC, but not ACA, primary cultures, cell proliferation was reduced after IR but not IGF1R knockdown.

**Conclusions:**

Overall, these data suggest that IGF1R localisation and IR expression represent new biomarkers predicting tumour aggressiveness, as well as possible molecular markers useful to patients’ stratification for more individualized IGF1R-IR targeted therapies or for novel pharmacological approaches specifically targeting IRA isoform.

**Supplementary Information:**

The online version contains supplementary material available at 10.1186/s12964-025-02115-0.

## Introduction

Adrenocortical carcinoma (ACC) is a rare endocrine tumour with a poor and heterogeneous prognosis, with a 5-years survival ranging from 13 to 80% [1, 2]. The estimated incidence is between 0.7 and 2 cases per million each year [3, 4]. The best curative option is the complete tumour resection [5, 6], but recurrences are common events [7]. First line medical treatment in advanced ACC is based on mitotane with or without the addition of etoposide, doxorubicin, and cisplatin (EDP) chemotherapy [5, 6]. However, the efficacy of these drugs is low and limited by severe adverse reactions [4, 8].

One of the most common molecular changes in ACC is the overexpression of the insulin-like growth factor 2 (IGF2), occurring in up to 90% of cases [9–13]. IGF2 is a key component of the complex IGF system that consists of three ligands (IGF1, IGF2, and insulin), three receptors (IGF1R, IGF2R, and the isoform A (IRA) and B (IRB) of the insulin receptor), six IGF-binding proteins (IGFBP1-6) and IGFBP related proteins (IGFBP-rPs) [14, 15].

The IGF2 role in ACC tumourigenesis has not been completely understood yet. Its involvement in ACC cells growth has been demonstrated by several in vitro studies in the preclinical gold standard H295R cell line [13, 16, 17]. Recently, new ACC cell lines have been established and characterized [18–20] with heterogeneous geno- and phenotypes that reflect the heterogeneity of ACC patients. However, experiments in mouse models suggest that IGF2 overexpression is not sufficient to trigger tumour development in the adrenal cortex, even in association with β-catenin activation [21, 22]. Furthermore, results about the correlation with ACC clinicopathological features are controversial. From one side, IGF2 showed no predictive value in ACC [10] and no correlation with clinicopathological, biological, and transcriptomic features [10, 11, 16] as well as relapse, metastasis and survival [10, 11]. On the contrary, another study reported that sporadic ACC patients with IGF2 overexpression had a 5-fold higher risk of recurrence [23].

These data provided the rationale for the use of IGF system inhibitors. Despite promising results have been obtained in preclinical models [10, 24], the phase III trial testing the dual IGF1R/IR inhibitor Linsitinib (OSI-906) in ACC showed a positive clinically response only in few patients [25]. This highlighted the need to better dissect the IGF pathway.

IGF2 is able to bind both IGF1 receptor and isoform A of the insulin receptor (IRA). IGF1R and IR are transmembrane receptor tyrosine kinases (RTKs) that share high degree of sequence and structural similarity. The alternative splicing of exon 11 of the insulin receptor (INSR) gene generates two IR splicing variants: the shorter one, lacking exon 11, named IRA and the long isoform IRB. IGF1R has high binding affinity for IGF1 and IGF2, and low affinity for insulin [26]. IRA is bound with high affinity by both insulin and IGF2, with consequent activation of mitogenic pathways, while IRB preferentially binds insulin and is involved in the regulation of metabolic functions [27].

The majority of studies investigating the IGF2 system in ACC focused on IGF1R. In particular, IGF1R expression was tested in ACC and compared to adrenocortical adenomas (ACA) and normal adrenals (NAG), with contrasting results. IGF1R was found more expressed in adult ACC respect to ACA [24, 28, 29] or NAG [30], but other studies reported no significant differences [10, 12, 31]. No correlation was found between IGF1R expression and clinicopathological features in adult ACC [12, 29]. However, in pediatric ACC, an overexpression of IGF1R was found compared to ACA [10] and a higher risk of metastases in childhood patients expressing high levels of IGF1R was demonstrated [10, 11]. Recently, an increased expression of IRA in ACC compared to normal adrenal tissue samples was found [12], supporting the hypothesis of a role played by IRA in mediating IGF2 promitotic effects. This agrees with observations made in other cancer types such as breast [32], prostate [33], endometrial [34], gestational trophoblastic neoplasia [35], ovarian [36], liver [37], lung [38], thyroid [39], and others where an increased expression of IR and/or a higher expression of IRA with respect to IRB was correlated with cancer development and progression [27].

The primary aim of this study was to determine the role of the IGF1R and IR in adrenocortical tumorigenesis. Specifically, we aim to investigate: (1) the IGF1R and IR expression and intracellular localisation in ACC and ACA; (2) the IR isoforms expression in ACC, ACA, and NAG and (3) IGF1R and IR involvement in mediating IGF2 tumourigenic effects in four different ACC cell lines and in primary cultured cells derived from surgically removed ACC and ACA.

## Materials and methods

### Patients and data collection

This is a retrospective European multicentre study conducted on behalf of the European Network for the Study of Adrenal Tumours (ENSAT). A total of 68 fresh-frozen adrenocortical tissues were collected at the University Hospital of Würzburg (Germany) and used for the evaluation of the isoforms A and B of the IR at mRNA levels. For the immunohistochemistry (IHC), 118 formalin-fixed, paraffin-embedded (FFPE) samples derived from primary or metastatic ACC tissues after tumour resection, of which 72 from University Hospital of Würzburg (Germany), 30 from the University of Florence (Italy), and 16 from Laiko Hospital Athens (Greece) were included. Moreover, 22 patients that underwent surgery because of ACA at Foundation IRCCS Ca’ Granda Ospedale Maggiore Policlinico were examined.

Clinical and pathological data on patients with ACC, including sex, age at diagnosis, tumour-related hormone excess at diagnosis, Ki67 proliferation index, ENSAT stage at first diagnosis, Weiss score and, S-GRAS were collected. The S-GRAS was calculated in patients after primary tumour resection as previously published [40] with a score ranging from 0 to 9. The date of last follow-up and status of patient (alive or date of death) were taken from medical records. Overall survival (OS) was defined as the time from primary tumour resection or diagnosis to death or last follow-up and was evaluated only for patients where primary tumour tissue was available. Progression-free survival (PFS) was evaluated as previously described and calculated also for recurrent or metastatic ACC [41]. The last follow-up was March 2024.

Moreover, clinical data including sex, age at diagnosis, and tumour-related hormone excess were collected also for patients with ACA.

The study was approved by the local ethics committees, and each patient gave written informed consent to the use of their tumour sample and clinical information.

### Immunohistochemistry

Slides of 4 μm thickness were obtained from representative formalin-fixed paraffin embedded (FFPE) blocks of primary adrenocortical tumours (118 ACC and 22 ACA). IHC staining with anti-IGF1R (G11 clone, Roche Diagnostics, Basel, CH; prediluted, EDTA buffer retrieval, #790–4346) and anti-IR (CT-3 clone, Santa Cruz Biotechnology, #sc-57342, RRID: AB_784102, CA, USA; 1:100, Citrate buffer retrieval) antibodies was performed using an automated stainer (BenchMark ULTRA, Ventana-Roche diagnostics, Oro Balley, AZ, USA), with diaminobenzidine revelation (Ultraview Universal DAB Detection Kit, Ventana-Roche diagnostics, #5269806001). Two independent investigators assessed the following parameters: percentage of positive tumoural cells, pattern of staining (i.e. cytoplasmic and/or membrane positivity) and intensity of staining and in case of discordant results a discussion between the two investigators was done until a consensus was reached. Immunoreactivities were graded manually using an optical microscope (Eclipse Ci-E, Nikon Instruments Inc., Tokyo, Japan) according to an immunoreactivity score (IRS) that is obtained by multiplying the percentage of positive cells (0–30% = 1; 31–60% = 2; 61–100% = 3) with the staining intensity (0 = absence of immunoreactivity, 1 = weak, 2 = medium intensity, and 3 = strong reactivity). Representative scans were captured via an Aperio AT-2 scanner (Leica Biosystems, Wetzlar, Germany).

### RNA isolation and quantitative Real-Time PCR (RT-qPCR) of adrenocortical tissues, ACC cell lines, and ACC and ACA primary cultures

IRA and IRB expression levels were analysed in 68 fresh-frozen adrenocortical tissues, including 14 NAG, 18 ACA, and 36 ACC, by quantitative real-time PCR (qRT-PCR) in a plex PCR system. Briefly, RNA was isolated from fresh frozen samples using the RNeasy Lipid Tissue Minikit (Qiagen, Hilden, Germany #74804) and the QuantiTect Reverse Transcription Kit (Qiagen #205311) was used for the reverse transcription. To differentiate the two isoforms IRA and IRB, we designed the amplification primer and the TaqMan hybridization probes reported in Supplemental Table 1 (IRA-IN and IRB-IN). We used β-actin (Hs9999903_m1) as housekeeping gene for normalization. Taqman real-time PCR was performed on a CFX96 (Bio-Rad Laboratories, Feldkirchen). RT-PCR contained 2 µL cDNA, 10 µL TaqMan Gene Expression Master Mix (Thermo Fisher Scientific), 0.2 µM primers and 0.1 µM probe in a final reaction volume of 20 µL. Temperature profiles were 50 °C for 2 min; 95 °C for 10 min, followed by 50 cycles of 95 °C for 15 s and 60 °C for 1 min. Expression levels were evaluated using Bio-Rad CFX Manager 2.0 software and normalized to those of β-actin using the ΔCT method. For the adrenocortical cell lines H295R, JIL-2266, MUC-1, and TVBF-7 the RNeasy Plus Mini Kit (Qiagen, Hilden, Germany #74136) was used to extract total RNA. For ACC and ACA tissues used for primary cell cultures, the frozen tissues (ACC, *n* = 4 and ACA, *n* = 8) were disaggregated with a Potter-Elvehjem support and Trizol (Ambion, Life Technologies Inc., Carlsband, CA, USA #15596018). The concentration and purity of total RNA were measured using NanoDrop Lite Spectrophotometer (Thermo Fisher Scientific, Whaltam, MA, USA) and RNA integrity was evaluated by 1% agarose gel electrophoresis. We used RevertAid H Minus First Strand cDNA Synthesis Kit (Thermo Fisher Scientific, Whaltam, MA, USA #K1632) to perform reverse transcription of a 1 µg of total RNA. Then, we carried out the RT-qPCR using the SsoFast™ EvaGreen^®^ Supermix (Bio-Rad Laboratories, Hercules, CA, USA #1725201), following the instructions of the manufacturer, in a QuantStudio™ 3 Real-Time PCR System (Thermo Fisher Scientific, Whaltam, MA, USA). Specific primers were designed for human IGF1R, IRA (IRA-F and IRA and IRB-R), and IRB (IRB-F and IRA and IRB-R) (Supplementary Table S1). The specificity of human IRA and IRB primers was previously validated [37], demonstrating high specificity for IRA and IRB detection, as well as equal amplification efficiency across all primer pairs. All reactions were performed in triplicate and media of Ct values was determined. Additionally, at least three experiments were performed for ACC cell lines. QuantStudioTM Design & Analysis Software was used. The ΔCt method was applied and the relative expression of single gene was expressed using the housekeeping GAPDH as reference gene.

### Cell cultures

Human ACC H295R cells were obtained from American Type Culture Collection (ATCC) (ATCC, Virginia, USA, #CRL-2128, RRID: CVCL_0458) and were grown in Dulbecco′s Modified Eagle′s Medium (DMEM)/Nutrient Mixture F-12 Ham (Sigma-Aldrich, Vermont, USA, #D84437) supplemented with 1% insulin-transferrin-selenium (ITS) + Premix (Corning, NY, USA, #354352), 2.5% Nu-Serum I (Corning, NY, USA, #355100), 10,000 U/ml penicillin, and 10,000 µg/ml streptomycin (Lonza group Ltd, Basel, CH, #DE17-602E). Cells were used for experiments with a maximum number of 20 passages. JIL-2266 cell line was cultured in 3:1 (v/v) DMEM-high glucose + Sodium Pyruvate (Gibco, Invitrogen, Life Technologies Inc., Carlsband, CA, USA, #41966-029) and F12 Nutrient Mixture (Ham; Invitrogen, #21765-029) modified as previously described [19] and was used for experiments with a maximum number of 30 passages. MUC-1 cell line was established as previously described [18] in DMEM advanced (Thermo Fisher Scientific, Massachusetts, USA, #12634-010) supplemented with 10% fetal bovine serum (FBS) (Gibco, Invitrogen, Life Technologies Inc., Carlsband, CA, USA, #A5256701), 10,000 U/ml penicillin and 10,000 µg/ml streptomycin. MUC-1 were used for experiments with a maximum number of 34 passages. TVBF-7 cell line was cultured in DMEM advanced supplemented with 10% FBS, 10,000 U/ml penicillin and 10,000 µg/ml streptomycin and 2mM L-glutamine (Sigma-Aldrich, Vermont, USA, #G7513) [20] and experiments were performed until a maximum number of 48 passages.

All cell lines have been authenticated using STR profiling at BMR Genomics Srl (1st July 2024). All experiments were performed with mycoplasma-free cells.

Fresh tissues were partly used to obtain primary cell cultures and partly stored at -80° C for nucleic acid and protein extraction. The study was approved by the local ethics committee and each patient gave informed consent to the use of his/her tumour sample and clinical information.

To establish primary cultures, freshly removed ACC (*n* = 4) and ACA (*n* = 8) tissues were subjected to mechanical and enzymatic digestion as described before [42]. Briefly, tissues were digested in DMEM (Sigma-Aldrich, Vermont, USA, #11965-092) with 2.5 mg/ml collagenase (Sigma-Aldrich, Vermont, USA, #C9891) at 37 °C for 2 h, passed on a 100 μm filter and cultured in DMEM supplemented with 20% FBS, 10,000 U/ml penicillin and 10,000 µg/ml streptomycin. When performing primary tumor cell cultures, contamination with non-tumoral cells was carefully minimized. Blood cells were removed in the days following culture initiation through repeated washing steps, adipocytes were eliminated after enzymatic digestion by centrifuging the cell suspension at 1000 rpm for 10 min, resulting in a lipid ring at the surface of the tube that was manually removed. Moreover, primary cultures were daily checked by visual inspection with an optical microscope to exclude fibroblast contamination.

### Western blot analyses

Total proteins extracted from cell lines and frozen tissues were quantified by BCA assay, separated on SDS/polyacrylamide gels and transferred to a nitrocellulose filter. Anti-IGF1R and anti-IR were used at 1:1000 (Cell Signalling Technology, Danvers, MA, respectively #3027, RRID: AB_2122378 and #3025, RRID: AB_2280448). GAPDH was used as housekeeping (Invitrogen, Life Technologies Inc., Carlsband, CA, USA, #AM4300, RRID: AB_2536381) at a dilution of 1:4000. Chemidoc-IT Imaging System (UVP, Upland, CA) was used to detect the chemiluminescence and densitometrical analyses were performed with the National Institutes of Health (NIH) ImageJ software (RRID: SCR_003070). Experiments were repeated at least three times.

### IGF2 ELISA assay

The quantitative determination of IGF2 was performed by evaluating the culture media of H295R, JIL-2266, MUC-1, and TVBF-7. Briefly, cells were seeded in a 6-well plate and the following day, cells were incubated with a serum free medium to avoid any growth factor contamination and make sure to measure the IGF2 secreted only by our cells. Finally, culture medium was collected and frozen at -80 °C until the day of the experiment (no more than 1 month). Sandwich ELISA assay was performed according to the datasheet instructions (LsBio, LifeSpan BioSciences, Inc., Seattle, WA, USA, #LS-F11731). In each experiment a standard dilution series was prepared and each sample was run in duplicate. The starved-based medium of each cell line was used as negative control. At least three experiments on different cell lines passages were conducted. The average zero standard optical density was subtracted from each standard point, control and samples and the following data analyses were performed with CurveExpert 1.4 software.

### Genetic silencing

To perform silencing of IGF1R we used SMARTpool siRNAs (Dharmacon, GE Healthcare Life Sciences, Chicago, IL, USA #L-003012-00-0020). Two different siRNAs against IR gene (Cat# 4392420, ID: s533803 and ID: s533802) were bought by Thermo Fisher Scientific (Waltham, Massachusetts, USA) and tested to choose the one that offered the best silencing efficiency (ID: s533803).

Subconfluent H295R cells, ACC (*n* = 4) and ACA (*n* = 8 primary cell cultures were transfected with IGF1R siRNA using Viromer Blue for miRNA/siRNA transfection reagent (Lipocalyx GmbH, Halle, D, #VB-01LB) and with IR siRNA using Lipofectamine RNAiMAX Transfection Reagent (Thermo Fisher Scientific, Massachusetts, USA, #13778150), according to manufacturer’s instructions. Moreover, H295R were transfected with IGF1R + IR siRNAs by Viromer Blue for miRNA/siRNA transfection reagent. Subconfluent JIL-2266, MUC-1, and TVBF-7 cells were transfected with IGF1R, IR, and IGF1R + IR siRNAs using Lipofectamine RNAiMAX Transfection Reagent. We conducted preliminary experiments to determine the optimal concentration of siRNAs and the silencing kinetics of IGF1R and IR alone and together. Based on these experiments, we selected a concentration of 25 pmol for IR siRNA and 37.5 nM for IGF1R siRNA, and an incubation time with siRNAs of 72 h. We used in each experiment a negative control siRNA, a non-targeting sequence without significant homology to the sequence of human, mouse or rat transcripts. Western blot was performed in parallel with each experiment to evaluate the silencing efficiency of IGF1R and IR. Only experiments achieving a silencing efficiency ≥ 70%, for both single and double silencing, were considered for analysis. In addition, due to the high homology between IGF1R and IR, the specificity of the siRNAs was tested by quantifying the mRNA levels of the specific target and the homologous receptor after silencing both IGF1R and IR at 24, 48 and 72 h. Experiments were repeated at least three times.

### Proliferation assay

Cell proliferation was assessed by colorimetric measurement of 5-bromo-2-deoxyuridine (BrdU) incorporation during DNA synthesis in proliferating cells as previously reported [42], according to the instruction of the manufacturer (Roche Diagnostics, Basel, CH, #11647229001). Cells were plated at the density of 1.4 × 10^4^ for H295R and ACC and ACA primary culture cells, 4.5 × 10^3^ for JIL-2266 and MUC-1 and 1.6 × 10^4^ for TVBF-7. Silencing of IGF1R, IR and IGF1R + IR was performed as described before. After 72 h of gene silencing, cells were incubated with starvation medium for 2 h and then were treated or not with Linsitinib 1µM (Selleck Chemicals, Houston, TX, USA, #S1091) in starvation medium for 24 h. BrdU was added to cell lines for 2 h and to ACC and ACA primary cultured cells for 24 h at 37 °C. All experiments with ACC cell lines were repeated at least three times and each determination was done in triplicate.

### Statistical analyses

Continuous variables were studied for their distribution. Parametric tests (e.g. T-test) were applied and mean ± standard deviation (SD) was used when a normal distribution was assumed. On the contrary, non-parametric tests and median and interquartile range (IQR) or median and 25th -75th percentile were applied in case of asymmetric variables. More in detail, to assess the significance between two series of data the non-parametric U of Mann-Whitney for independent or Wilcoxon test for dependent data were applied. To evaluate significance between more than two groups of data, the non-parametric Kruskal Wallis one-way ANOVA test with Dunn’s post hoc tests was used. Correlation between two continuous variables was done by Pearson or Spearman correlation depending on the distribution of the variable. Discrete and categorical variables were described as number and percentage and compared through the chi-squared or Fisher’s exact tests, when appropriate. Univariate and multivariate logistic regression analyses were performed to evaluate the risk of IGF1R and/or IR expression and localisation in determining Ki67 ≥ 10 and Weiss score ≥ 6. Kaplan–Meyer method was used to describe the overall survival (OS) and PFS according to IR and IGF1R expression and localisation. The log-rank test was used to test the difference in survival across groups.

Calculations were performed by IBM SPSS statistics, version 29.0.1.1 (SPSS Inc., Chicago, IL, USA, RRID: SCR_016479) and GraphPad Prism 9.0 software (GraphPad Software, Inc., La jolla, CA, RRID: SCR_002798). *p* < 0.05 was accepted as statistically significant.

## Results

### IGF1R and IR immunostaining and localisation and their association with clinicopathological features

The IGF1R and IR expression and localisation were investigated by IHC in a cohort of patients with ACC (*n* = 118), including both primary tumours (*n* = 107) and local recurrences or distant metastases (*n* = 11), and ACA (*n* = 22) and association with clinicopathological features was assessed. Demographic and clinical characteristics are detailed in Supplementary Table S2.

**Fig. 1:**
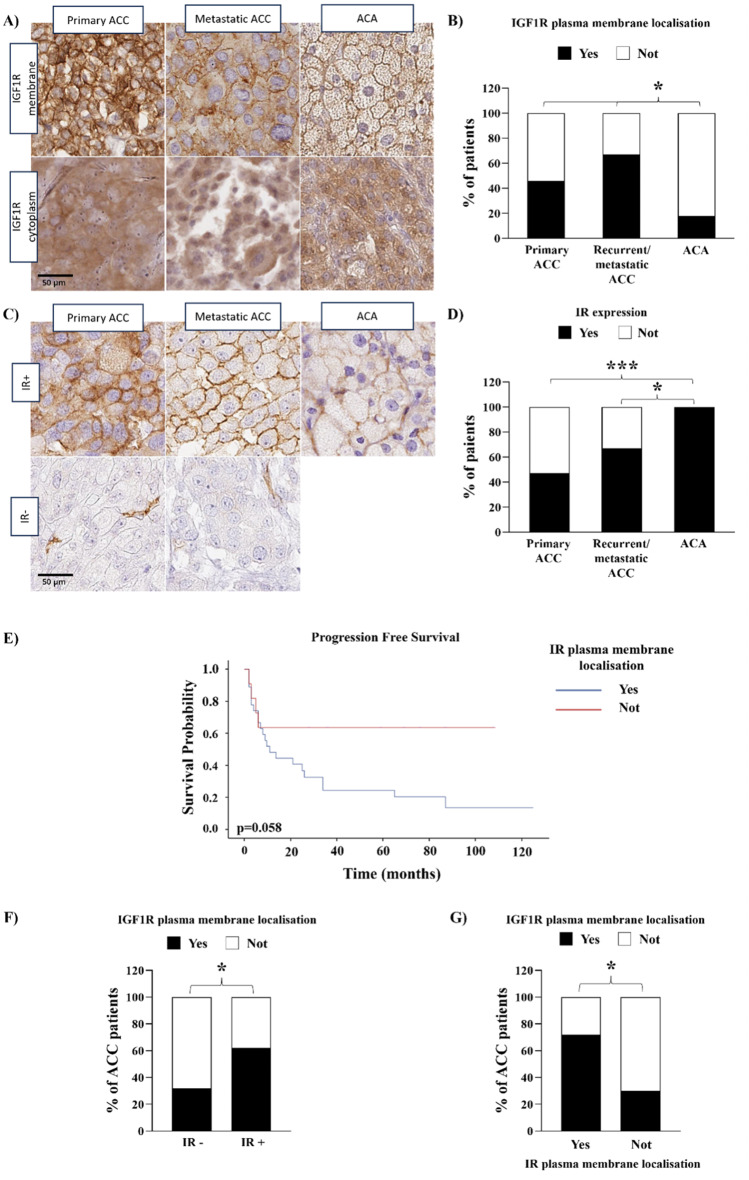
IGF1R and IR expression and localisation in ACC and ACA. **A**) Composite panel depicting representative cases of primary and metastatic ACC and ACA showing either presence or absence of IGF1R expression on the cell membrane. **B**) The stacked bar chart shows the percentage of patients with only or at least IGF1R plasma membrane localisation (black bars) and IGF1R other cellular localisations (cytoplasm and/or nucleus, white bars) in primary ACC (*n* = 90), recurrent/metastatic ACC (*n* = 9), and ACA (*n* = 17). *=*p* < 0.05, chi-squared test. **C**) Composite panel showing representative cases of primary and metastatic ACC and ACA, featuring either expression or negativity for insulin receptor (internal positive control represented by endothelial cells). **D**) The graph shows the percentage of patients with IR expression (black bars) in primary ACC (*n* = 92), recurrent/metastatic ACC (*n* = 9), and ACA (*n* = 19). *=*p* < 0.001, chi-squared test. **E**) Primary ACC with IR plasma membrane localisation shows a worse progression free survival. Kaplan-Meier analysis of the progression free survival in patients with IR plasma membrane localisation (*n* = 27) and in those without plasma membrane localisation (*n* = 11). **F**&**G**) IGF1R plasma membrane localisation is associated with IR expression and plasma membrane localisation. The bar charts show the association between the percentage of ACC patients with only or at least IGF1R plasma membrane localisation and the IR expression (**F**) (*n* = 76; “IR-” = no immunoreactivity to IR antibody, “IR+” = IR expression) or the IR plasma membrane localisation (**G**) (*n* = 39). *=*p* < 0.05, chi-squared test

IGF1R was expressed in 85.7% of primary ACC, in 81.8% of recurrent/metastatic ACC and in all ACA. Moreover, the IRS of IGF1R was higher in ACA than primary ACC (4.0 (2.0–6.0) vs. 2.0 (1.0–4.0), *p* < 0.05, respectively), but no differences were found with respect in recurrent and metastatic ACC. Interestingly, a significantly different IGF1R intracellular localisation between ACC and ACA was observed (Fig. 1A&B). In particular, IGF1R was localized at the plasma membrane in a higher percentage of both primary and recurrent/metastatic ACC compared to ACA (41/90 in primary ACC; 6/9 in recurrent or metastatic ACC and 3/17 in ACA, *p* < 0.05) (Fig. 1B). However, no significant difference was observed between primary and recurrent/metastatic ACC (Fig. 1B). 46.7% of primary ACC and 66.7% of recurrent or metastatic tissues expressed IR whereas all ACA patients had a positive staining (Fig. 1C&D). In addition, a higher IR IRS was found in ACA compared to primary ACC (*p* < 0.01). No difference in terms of IR localisation was found between the groups (plasma membrane localisation of IR in 74.4% of primary ACC, 83.3% of recurrent or metastatic ACC, and 84.2% of ACA).

We evaluated the correlation between IGF1R and IR expression and localisation and the clinicopathological features in the primary ACC group. No association between IGF1R expression and any other variables was found. However, patients with high IGF1R expression (> 70% of positive cells) had a higher Ki67 (*p* < 0.05) (Table 1). Interestingly, IGF1R plasma membrane localisation was associated with a higher Ki67 index (*p* < 0.01) and Weiss score (*p* < 0.001) (Table 1). Analysing the single nine histological criteria of the Weiss score, we found that patients with IGF1R plasma membrane localisation were characterized by a higher mitotic rate (*p* < 0.05), an increased presence of atypical mitoses (*p* < 0.05), and venous invasion (*p* < 0.01) (Supplementary Table S3).

**Table 1:** Clinicopathological features in low (≤ 70% of positive cells) and high (> 70% of positive cells) IGF1R ACC groups and in those with or without IGF1R membrane localisation

	Low IGF1R (*n* = 82)	High IGF1R (*n* = 23)	*p* value	IGF1R plasma membrane localization (*n* = 41)	IGF1R no plasma membrane localization (*n* = 49)	*p* value
Female (%)	58.5	78.3	0.08*	73.2	55.1	0.08*
Age	51.43 ± 13.65	44.35 ± 17.95	***0.04*** ^***+***^	49.6 ± 16.4	48.9 ± 14.3	0.84^+^
≥ 50 yrs (%)	56.1	34.8	0.07*	53.7	49.0	0.66*
ENSAT stage (I-II) (%)	49.4	60.9	0.33*	51.2	50.0	0.91*
ENSAT stage (III-IV) (%)	50.6	39.1		48.8	50.0	
Ki67	15.0 (13)	20.0 (28)	0.36^^^	20.0 (16)	10.0 (15)	***< 0.001*** ^***^***^
0 < Ki67 < 9 (%)	24.1	30.0	0.04*	5.3	37.5	0.002*
10 < Ki67 < 19 (%)	39.2	10.0		36.8	29.2	
Ki67 ≥ 20 (%)	36.7	60.0		57.9	33.3	
Weiss score	5.9 ± 1.6	5.3 ± 1.9	0.17^+^	6.6 ± 1.5	5.3 ± 1.7	***< 0.001*** ^***+***^
Weiss score < 6 (%)	38.8	55.6	0.20*	17.6	60.0	< 0.001*
Weiss score ≥ 6 (%)	61.2	44.4		82.4	40.0	
S-GRAS 0–1 (%)	14.6	7.7	1.00^−^	5.3	20.0	0.27^−^
S-GRAS 2–3 (%)	41.5	46.2		52.6	33.3	
S-GRAS > 3 (%)	43.9	46.2		42.1	46.7	
Secreting tumours (%)	63.4	70.0	0.58*	62.9	74.4	0.27*
Non-secreting tumours (%)	36.6	30.0		37.1	25.6	

*p value obtained from chi-squared test, ^+^p value obtained from T-test, ^-^p value obtained from Fisher exact test, ^^^p value obtained from Mann Whitney test

Since a higher Ki67 was found in both patients with high IGF1R expression and in those with plasma membrane localisation, we analysed their impact in determining Ki67 ≥ 10 in the primary ACC cohort by logistic regression. Univariate analysis showed that patients with IGF1R membrane localisation were approximately ten times more likely to have a Ki67 ≥ 10 (OR = 10.8; 95% CI [2.3–50.3], *p* < 0.01) (Supplementary Table S4) and this odd was maintained in multivariate analysis considering as variables also IGF1R expression (OR = 14.5; 95% CI [2.8–74.9], *p* < 0.01) (Supplementary Table S4). No differences were found among groups in term of overall and progression-free survival.

Regarding IR, we found that patients with IR expression (IR+) had a higher ENSAT stage (*p* < 0.05), Ki67 index (*p* < 0.01) and Weiss score (*p* < 0.05) compared to patients with negative IR staining (IR-) (Table 2). In agreement, a positive correlation was found between the percentage of IR + cells and both Ki67 (Spearman’s Rho = 0.41, *p* < 0.001) (Supplementary Figure S1A) and Weiss score (Spearman’s Rho = 0.32, *p* < 0.01) (Supplementary Figure S1B). In addition, as for IGF1R, IR plasma membrane localisation was associated with higher Ki67 index and Weiss score (*p* < 0.05) (Table 2). It is of interest to note that having IR expression constitutes a greater risk of having a Ki67 ≥ 10 (OR = 4.2; CI [1.3–12.8], *p* < 0.05) and Weiss score ≥ 6 (OR = 3.5, 95% CI [1.3–9.5], *p* < 0.05) compared to not expressing IR in primary ACC (Supplementary Table S5). Furthermore, among patients expressing IR, those with plasma membrane localisation have a further increased risk of having both Ki67 ≥ 10 (OR = 16.6, 95% CI [1.5–172], *p* < 0.05) and Weiss score ≥ 6 (OR = 7.0, 95% CI [1.1-44.06], *p* < 0.05) respect to patients without IR plasma membrane localisation (Supplementary Table S5). Accordingly, although statistical significance was not reached, a trend toward worse progression free survival in patients with IR plasma membrane localisation respect to those without IR plasma membrane localisation was observed (*p* = 0.058) (Fig. 1E).

**Table 2:** Clinicopathological features in ACC with or without expression of IR and in those with or without IR plasma membrane localisation

	IR- (*n* = 49)	IR+ (*n* = 43)	*p* value	IR plasma membrane localisation (*n* = 32)	IR no plasma membrane localisation (*n* = 11)	*p* value
Female (%)	59.2	69.8	0.29*	75.0	54.5	0.26^−^
Age	51.9 ± 14.0	49.2 ± 15.6	0.52^+^	48.7 ± 14.6	50.6 ± 19.0	0.73^+^
≥ 50 yrs (%)	57.1	48.8	0.42*	50.0	45.5	0.79*
ENSAT stage (I-II) (%)	62.5	37.2	0.02*	37.5	36.4	1.00^−^
ENSAT stage (III-IV) (%)	37.5	62.8		62.5	63.6	
Ki67	10.0 (15)	20.0 (18)	***< 0.001*** ^***^***^	20.0 (15)	10.0 (34)	0.13^^^
0 < Ki67 < 9 (%)	37.0	12.2	0.009*	3.3	36.4	***0.02*** ^***−***^
10 < Ki67 < 19 (%)	34.8	31.7		36.7	18.2	
Ki67 ≥ 20 (%)	28.3	56.1		60.0	45.5	
Weiss score	5.4 ± 1.6	6.4 ± 1.5	***0.003*** ^***+***^	6.0 (2.0)	5.0 (4.0)	0.26^^^
Weiss score < 6 (%)	53.7	25.0	0.01*	16.0	57.1	***0.047*** ^−^
Weiss score ≥ 6 (%)	46.3	75.0		84.0	42.9	
S-GRAS 0–1 (%)	19.0	5.0	0.37	7.1	0.0	1.00^−^
S-GRAS 2–3 (%)	38.1	50.0		50.0	50.0	
S-GRAS > 3 (%)	42.9	45.0		42.9	50.0	
Secreting tumours (%)	60.5	60.0	0.96*	37.9	45.5	0.73^−^
Non-secreting tumours (%)	39.5	40.0		62.1	54.5	

*p value obtained from chi squared, ^+^p value obtained from T-test, ^-^p value obtained from Fisher exact test, ^^^p value obtained from Mann Whitney test

Lastly, an association between IGF1R plasma membrane localisation and both IR expression and plasma membrane localisation was found (*p* < 0.05) (Fig. 1F&G).

### mRNA expression of IRA and IRB in human adrenocortical tissues

Since IHC analysis does not distinguish the two isoforms of IR, as antibodies able to discriminate IRA from IRB are not currently available, we performed real time qPCR (RT-qPCR) using IRA or IRB specific primers in 36 ACC, 18 ACA, and 14 NAG.

Clinical and pathological characteristics of the patients evaluated by qRT-PCR are summarized in Supplementary Table S6.

Considering the entire cohort of 68 patients (NAG, ACA, and ACC), IRA mRNA expression positively correlated with IRB (*r* = 0.63, *p* < 0.0001). Similar results were observed also when considering ACC and ACA separately (*r* = 0.79, *p* < 0.0001 and *r* = 0.83, *p* < 0.0001, respectively), whereas only a trend was found within the NAG (*r* = 0.49, *p* = 0.08) (Supplementary Figures S2).

**Fig. 2:**
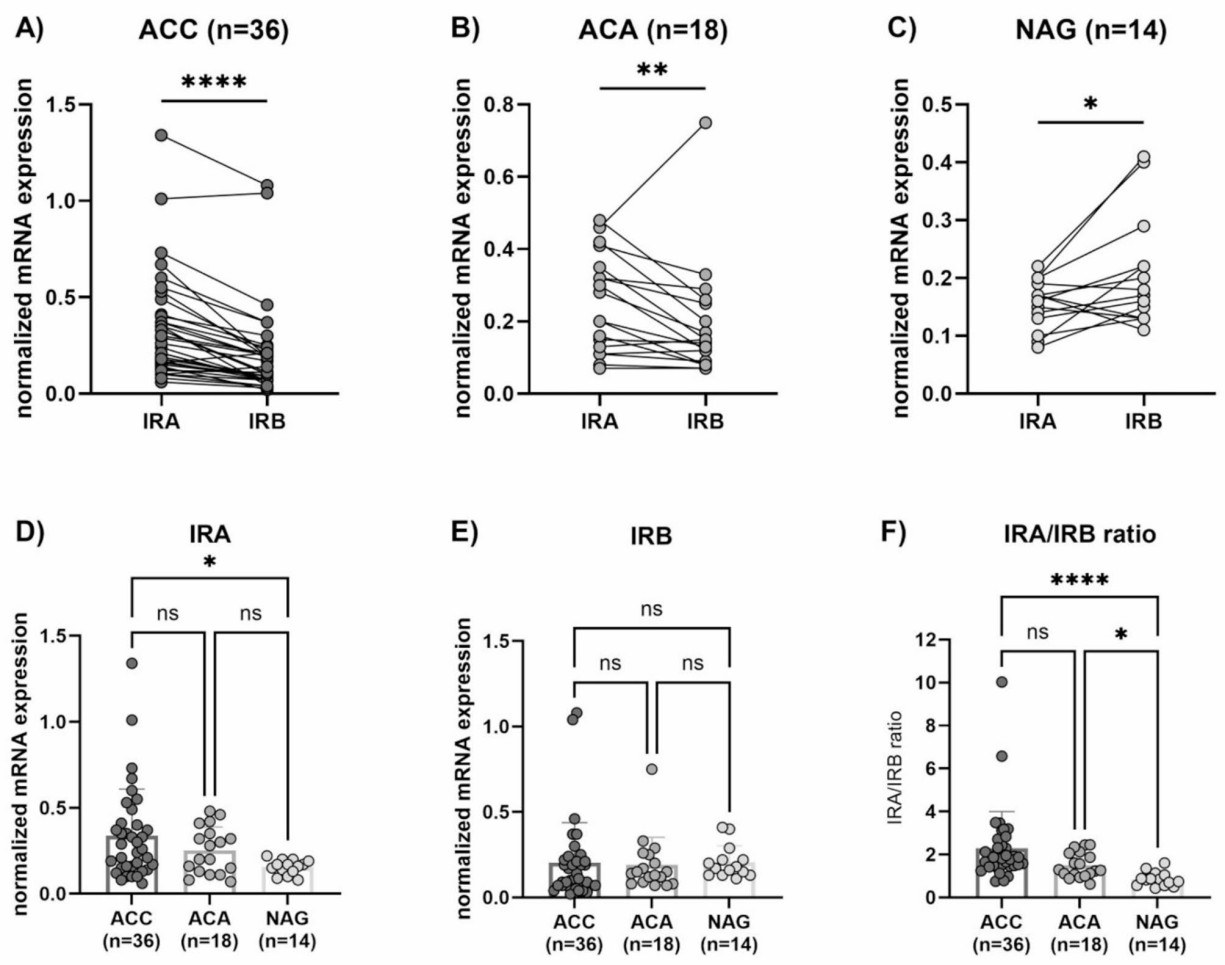
Normalized IRA and IRB mRNA levels in ACC, ACA and NAG. IRA and IRB mRNA levels within the same sample in (**A**) adrenocortical carcinoma (ACC) (*n* = 36), (**B**) adrenocortical adenoma (ACA) (*n* = 18), and (**C**) normal adrenal gland (NAG) (*n* = 14). Difference in IRA (**D**), IRB (**E**) and IRA/IRB ratio (**F**) among the three groups

Within the same sample, IRA expression was significantly higher than IRB in both ACC (median IRA 0.27 (0.25) vs. IRB 0.12 (0.15), *p* < 0.0001, Fig. 2A) and ACA (IRA 0.24 (0.24) vs. IRB 0.14 (0.16), *p* = 0.001, Fig. 2B). On the contrary, in NAG IRA was significantly lower than IRB (0.16 (0.07) vs. 0.17 (0.11), *p* = 0.03, Fig. 2C). IRA levels were significantly higher in ACC compared to NAG (*p* = 0.02) but not to ACA (Fig. 2D), whereas no differences were observed in IRB expression among the three groups (Fig. 2E). Interesting, IRA/IRB ratio was higher in tumours (1.884 (1.15) in ACC and 1.27 (1.03) in ACA) than normal tissues (0.83 (0.51), *p* < 0.0001 and *p* = 0.015 compared to ACC and ACA, respectively; Fig. 2F).

### Evaluation of IGF system in human ACC cell lines

**Fig. 3:**
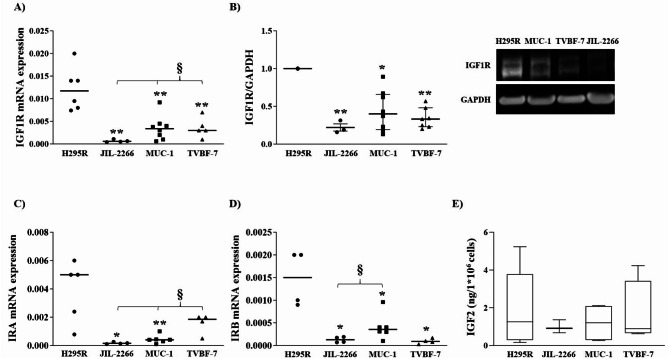
Insulin-like growth factor (IGF) system in H295R, JIL-2266, MUC-1, and TVBF-7. IGF1R mRNA (**A**) and protein (**B**) expression in ACC cell lines. **A**) The vertical scatter plot shows the normalized IGF1R mRNA expression for each cell line. GAPDH was used as reference gene. Horizontal bars represent median (IQR) values. ** = *p* < 0.01 vs. H295R; §= *p* < 0.05 vs. JIL-2266. **B**) On the left the result of the densitometrical analyses of at least three experiments (median (IQR)). IGF1R was normalized on GAPDH. On the right representative immunoblot. The membranes were incubated with IGF1R antibody and then reprobed with anti-GAPDH antibody. JIL-2266, MUC-1 and TVBF-7 were normalized on H295R. * = *p* < 0.05; ** = *p* < 0.01 vs. H295R. **C**&**D**) The charts illustrate the different IR isoforms mRNA expression between H295R, JIL-2266, MUC-1, and TVBF-7. Horizontal bars represent median values. * = *p* < 0.05; ** = *p* < 0.01 vs. H295R and § = *p* < 0.05 vs. JIL-2266. **E**) IGF2 secretion in culture media measured by ELISA assay in H295R, JIL-2266, MUC-1, and TVBF-7. Cells were incubated for 2 h with a serum-deprived cell culture media and, in case of H295R, also with insulin-transferrin-selenium (ITS) + Premix deprivation. Starved base medium of each cell line was used as negative control. Secretion was expressed as ng of IGF2 secreted by 1 * 10^6^ cells

We evaluated the expression of IGF1R and IR isoforms in a panel of four different cell lines: H295R and JIL-2266, derived from ACC primary tumours of female patients, MUC-1 and TVBF-7, derived from ACC distant and perirenal lymph-node metastasis of male patients, respectively. We found a great variability of receptor expression that reflects the heterogeneity found in ACC samples. H295R cells had a higher expression of IGF1R, both at transcript and protein levels, than JIL-2266, MUC-1 and TVBF-7 (Fig. 3A&B). Greater levels of IRA and IRB transcripts were measured in H295R in comparison with JIL-2266 and MUC-1 while only higher IRB levels were found in H295R respect to TVBF-7. (Figs. 3C&D). Furthermore, as in ACC group, RT-qPCR revealed a higher expression of isoform A than B in H295R, with an IRA/IRB ratio of 3.27 ± 1.17, and a strong unbalance toward IRA with an IRA/IRB ratio of 17.53 ± 5.34 in TVBF-7. On the contrary, JIL-2266 and MUC-1 showed a small but almost equal amount of both isoforms (Fig. 3C&D).

All cell lines secreted IGF2, without any significant differences between them (Fig. 3E).

### IGF1R and IR role in IGF2-mediated proliferation in ACC cell lines

To examine the role of IGF1R and IR in mediating IGF2 effects on cell proliferation, we used two different experimental strategies: (1) we performed IGF1R and IR single and double gene silencing, incubating cells with IGF1R and/or IR siRNAs and (2) we used Linsitinib to inhibit both receptors.

Due to the high homology between IGF1R and IR, we first evaluated the specificity of siRNAs by mRNA quantification of the specific target and of the homologous receptor, after both IGF1R and IR silencing (Supplementary Figure S3). It is worth noting that IR siRNA recognizes both IRA and IRB isoforms and that no specific siRNAs against one or the other isoform are available.

**Fig. 4:**
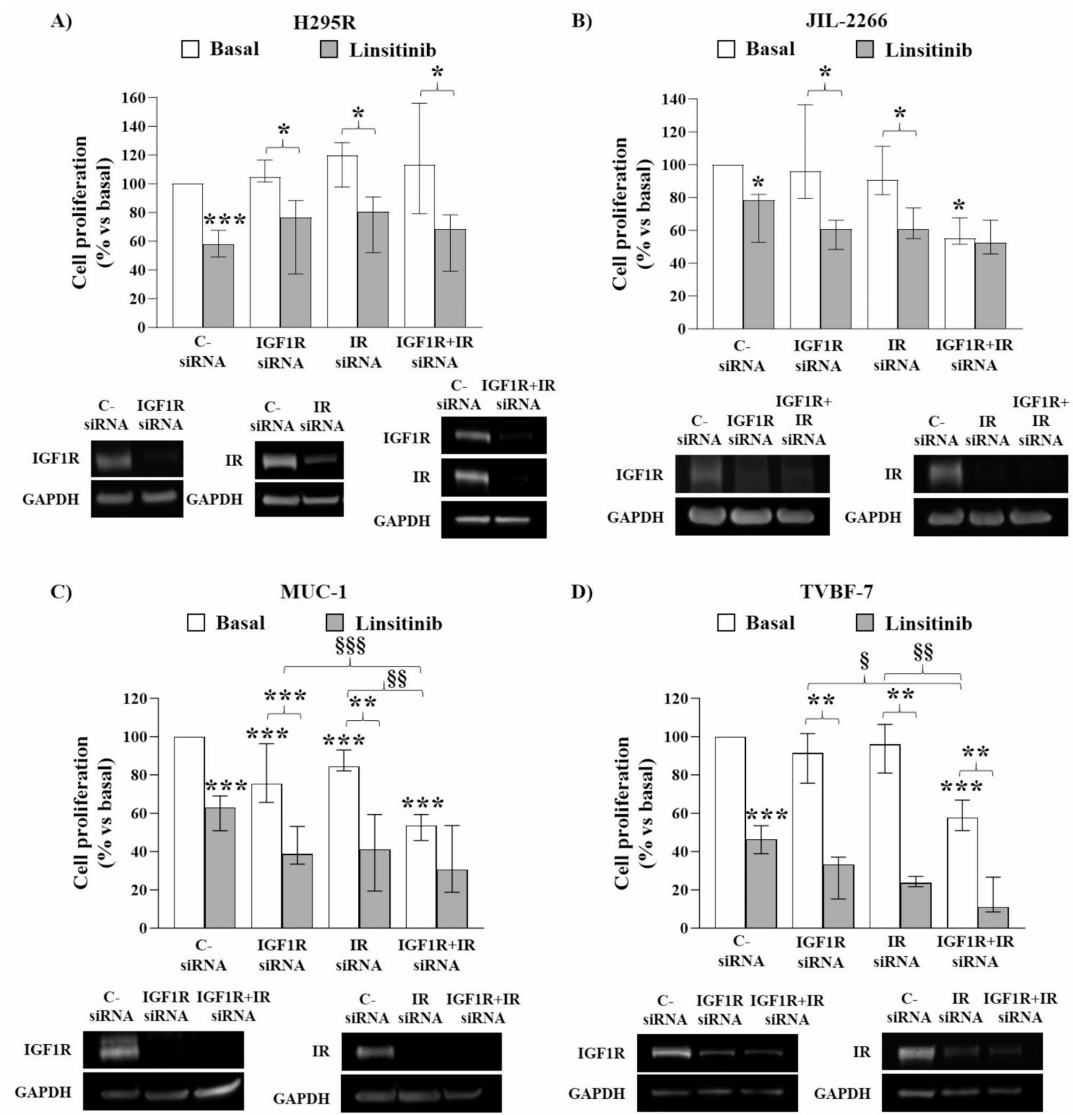
Effect of IGF1R and/or IR gene knockdown or Linsitinib inhibition on proliferation in human ACC cell lines. H295R (**A**), JIL-2266 (**B**), MUC-1 (**C**) and TVBF-7 (**D**) proliferation assays. Cells were seeded at the density of 1.4 × 10^4^ for H295R, 4.5 × 10^3^ for JIL-2266 and MUC-1 and 1.6 × 10^4^ for TVBF-7 in a 96-well. The following day they were silenced for IGF1R, IR, or IGF1R + IR for 72 h and then, a serum starvation for 2 h followed by incubation with Linsitinib 1µM for 24 h was performed. BrdU was added for 2 h and its incorporation in newly synthesized DNA was measured. Western blot was performed in parallel with each experiment to evaluate the silencing efficiency of IGF1R and IR and only experiments achieving a silencing efficiency ≥ 70% were considered for analysis. Experiments were repeated at least 3 times and each determination was done in triplicate. Graphs show the median and IQR expressed as percentage of basal C- siRNA of cell proliferation in H295R (**A**), JIL-2266 (**B**), MUC-1 (**C**) and TVBF-7 (**D**). * = *p* < 0.05; ** = *p* < 0.01; *** = *p* < 0.001 vs. c- siRNA; § = *p* < 0.05; §§ = *p* < 0.01; §§§ = *p* < 0.001. Below each graph representative immunoblots are present to illustrate the efficiency of cell silencing after IGF1R, IR and IGF1R + IR knockdown in H295R (**A**), JIL-2266 (**B**), MUC-1 (**C**) and TVBF-7 (**D**)

We observed that H295R cell proliferation was not affected by either single IGF1R or IR nor double IGF1R + IR silencing (Fig. 4A). On the contrary, silencing of both receptors reduced cell proliferation in JIL-2266 (-44.95 (16.04)%, *p* < 0.05 vs. control cells), MUC-1 (-46.31 (13.58)%, *p* < 0.001 vs. control cells), and TVBF-7 cell lines (-42.12 (15.89)%, *p* < 0.001 vs. control cells) (Fig. 4B-D). Moreover, MUC-1 proliferation was also lowered after the knockdown of IGF1R alone (-24.58 (30.70)%, *p* < 0.001 vs. control cells) or IR alone (-15.46 (10.94)%, *p* < 0.001 vs. control cells) (Fig. 4C).

Linsitinib incubation was able to inhibit cell proliferation of all ACC cell lines in basal condition (-42.22 (18.47)%, *p* < 0.001 vs. control cells in H295R; -21.56 (29.23)%, *p* < 0.05 vs. control cells in JIL-2266; -36.97 (18.16)%, *p* < 0.001 vs. control cells in MUC-1; -53.5 (14.6)%, *p* < 0.001 vs. control cells in TVBF-7) (Fig. 4A-D). As expected, the dual IGF1R-IR inhibitor maintained its antiproliferative effect after single receptor silencing in all cell lines, but not in the absence of both receptors in JIL-2266 and in MUC-1. On the contrary, Linsitinib retained an antiproliferative effect after double silencing in H295R (-44.66, *p* < 0.05 vs. double silenced cells) and TVBF-7 (-46.69, *p* < 0.01 vs. double silenced cells) (Fig. 4A-D).

### IGF1R and IR role in IGF2-mediated proliferation in ACC and ACA primary cultured cells

**Fig. 5:**
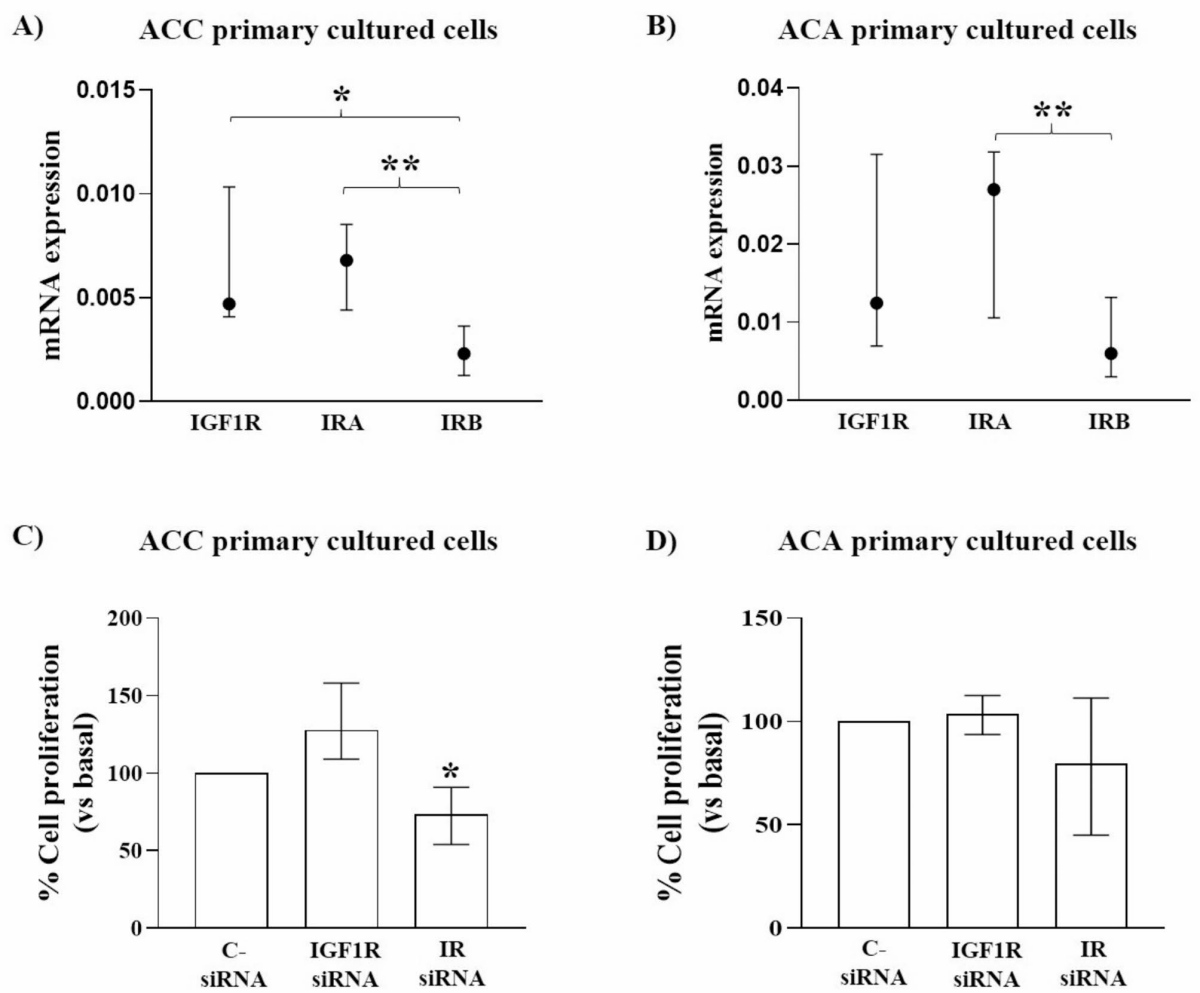
IGF system in ACC and ACA primary cultures. **A**&**B**) IGF1R, IRA, and IRB expression in ACC (*n* = 4) (**A**) and ACA (*n* = 8) (**B**) primary cultures. The graphs show ΔCt of genes normalized on GAPDH with median and IQR. *=*p* < 0.05 **C**&**D**) 1.4 × 10^4^ ACC (*n* = 4) and ACA (*n* = 8) primary cultured cells were seeded and silenced the following day for IGF1R or IR for 72 h. At the end an incubation with a serum-free media for 24 h followed by addition of BrdU for additional 24 h was performed. Each determination was done at least in triplicate. The charts values represent median and IQR expressed as percentage of cell proliferation respect to basal C- siRNA. *=*p* < 0.05 vs. C- siRNA

Silencing experiments were replicated in ACC (*n* = 4) and ACA (*n* = 8) primary cultures. A statistically significant higher expression of IRA than IRB was observed in ACC (*p* < 0.01) (Fig. 5A) and ACA (*p* < 0.01) (Fig. 5B), with an average IRA/IRB ratio of 2.90 ± 1.07 for ACC and 3.52 ± 1.46 for ACA. In both groups of tumours, no significant variation of cell proliferation after IGF1R knockdown was found. On the contrary, a significant reduction of growth rate was observed in IR silenced cells in ACC (-26.34 (36.97)%, *p* < 0.05 vs. control cells), but not in ACA (Fig. 5C&D).

## Discussion

The present study demonstrated an association of IGF1R and IR plasma membrane localisation, as well as IR expression, with a worse tumour behaviour in ACC. Moreover, it firstly proved the involvement of IR in mediating IGF2 proliferative effects in ACC cells.

Although in ACC the overexpression of IGF2 occurred in 90% of patients and its involvement in ACC cell growth has been demonstrated in H295R cell line [13, 16, 17], the IGF2 role in ACC tumourigenesis has not been completely understood yet. Moreover, the majority of studies investigating the IGF2 system in ACC focused on IGF1R, without considering the isoform A of IR, which has a high affinity for IGF2.

We performed IHC analysis of IGF1R and IR on a large cohort of ACC (*n* = 118) comparing the results with that obtained in ACA (*n* = 22). Regarding IGF1R, we found that IRS was higher in ACA than in primary, but not in recurrent or metastatic ACC. However, IGF1R was more frequently localized at the plasma membrane in ACC than in ACA, suggesting an increase of receptors pool available for IGF2 binding at the cell surface in ACC. To the best of our knowledge, this is the first study that investigates the cellular localisation of IGF1R in adrenal tumours, whereas the investigation of IGF1R expression in ACC and ACA has been a widely explored topic. Either a similar expression of IGF1R in ACC and ACA [10, 31] or an increased IGF1R in ACC with respect to ACA [24, 28, 29] were described. As for IR, we found no immunoreactivity in approximately half of ACC cases, whereas all ACA showed IR expression, with a higher IRS in respect with primary ACC, but a comparable intracellular localisation. Only one previous study investigated IR protein expression in adrenal tumours, showing no differences in positivity to IR staining, but a higher IR intensity in ACC than ACA [31].

A possible explanation for these discrepancies may be due to the specificity of the antibodies used for IHC, since IGF1R and IR display a high degree of homology. In the present study, we selected two antibodies against IGF1R and IR, whose specificity and absence of cross-reactivity with the homologous receptor have been verified and guaranteed. In addition, it is worth noting that the cohort of subjects analysed in this study is larger than in the previously published papers.

Then, we evaluated the association between the expression and localisation of the two RTKs and the clinicopathological features in primary ACC. No association has been found between IGF1R expression and the clinicopathological features in ACC, as previously reported [12, 29]. Only in a cohort of paediatric ACC patients, a correlation was observed with metastasis [11]. Interestingly, we found that IGF1R plasma membrane localisation was associated with a worse tumour behaviour, consistently with a hyper activation of the IGF2 mediated promitogenic pathways triggered by an increased availability of IGF1R at the cell surface, where it binds IGF2 to mediate downstream pathways. Specifically, in patients with IGF1R plasma membrane localisation, a high Ki67 index and total Weiss score was found respect to patients without IGF1R plasma membrane localisation. Additionally, among individual Weiss parameters, a high number of mitoses, presence of atypical mitoses and venous invasion were observed in patients with IGF1R plasma membrane localisation compared with patients without it. On the contrary, a high IGF1R expression was associated only with a high Ki67 index. The result of the multivariate logistic regression demonstrated a predictive role for IGF1R plasma membrane localisation in determining a Ki67 index higher than 10, emphasizing that the localisation of IGF1R on the plasma membrane is more important for tumour progression than its overall expression.

A positive IR staining was associated with a higher ENSAT stage, Ki67 index, and Weiss score. Accordingly, a positive correlation between the percentage of cells positive to IR and both the Ki67 index and Weiss score was observed. Furthermore, among samples with IR expression, those having the receptor localised on the plasma membrane were associated with even higher Ki67 index and Weiss score and appear to show a trend toward a worse PFS compared with patients without IR plasma membrane localisation. In agreement, univariate logistic regression showed a predictive role of both IR expression and plasma membrane localisation in determining a high Ki67 index and Weiss score. It is of interest to note that IGF1R plasma membrane localisation was associated with both IR expression and its plasma membrane localisation. This finding implies that when IGF1R is localised on the plasma membrane, there is a higher likelihood of IR also being expressed and localized on the plasma membrane, suggesting a significant interplay between these two receptors in tumour progression. This co-localisation may indicate a synergistic effect, potentially enhancing the IGF2 dependent signalling pathways that contribute to aggressive tumour behaviour. Future studies involving larger, independent cohorts of ACC patients are warranted to validate these findings and enhance their clinical applicability.

Nevertheless, IHC analysis on IR expression does not take into account the expression of the two different isoforms of IR, IRA and IRB, that display different binding properties and lead to different downstream responses [27, 43]. The analysis revealed that IRA was the most abundant isoform in both ACC and ACA, while IRB was prevalent in NAG. Moreover, the IRA/IRB ratio was higher in tumours than in normal adrenal tissues. Our results agree with previously published data demonstrating a greater expression of IRA in ACC than in NAG [12]. Additionally, they highlight a similar scenario for ACC and ACA, an aspect that, to the best of our knowledge, has never been reported before. This result suggests that the alteration of IR alternative splicing may play a role in the tumourigenesis of both adrenal adenomas and carcinomas. We can hypothesize that IRA overexpression may have a stronger impact in carcinomas, that also overexpress its ligand IGF2, than in adenomas.

With the purpose to evaluate the specific contribution of IGF1R and IR in mediating ACC cell proliferation, we performed in vitro experiments with four different cell lines: H295R and JIL-2266, derived from ACC primary tumours, and MUC-1 and TVBF-7 derived from ACC metastatic tissues [18–20].

The characterization of the IGF2 pathway showed that these cell lines secrete similar amounts of IGF2 in the culture medium, express IGF1R, that is particularly abundant in H295R, and display an unbalance of the IR isoforms towards IRA (H295R and TVBF-7) or a similar expression of both isoforms (JIL-2266 and MUC-1). IGF2 secretion in H295R in cell culture media has been previously demonstrated [17] whereas data in the other cell lines were not available up to now.

To assess the single contribution of IGF1R and IR in mediating IGF2 effects on cell proliferation we performed specific genetic silencing followed by proliferation assays. In H295R cells neither IGF1R or IR silencing nor the double silencing impaired cell proliferation, suggesting a possible involvement of other components of the complex IGF system in mediating IGF2 mitogenic effects, such as IGF2R, a scavenger receptor that acts as a clearance receptor for IGF2, but can also play an oncogenic role [44, 45]. In addition, Linsitinib was able to reduce cell growth regardless of the presence of IR and IGF1R, further supporting the involvement of other mechanisms. Exploring the interplay between IGF1R, IR, and other components of the IGF system, will be essential to better understand the compensatory mechanisms that may drive tumor growth in the absence of IGF1R or IR activity. Nevertheless, we have to take into consideration that H295R has both TP53 and CTNNB1 mutations [20], both connected with survival and growth of the tumor that could have impacted on the result obtained. Conversely, the experiments performed in JIL-2266, MUC-1, and TVBF-7 revealed a decrease of cell proliferation after IGF1R + IR knockdown, suggesting a contribution of both IGF1R and IR. Only in MUC-1 cells, the single receptor silencing was sufficient to reduce cell growth. A possible explanation is that in JIL-2266 and TVBF-7 the absence of one receptor could be compensated by the presence of the other, as previously demonstrated [46]. All cell lines analysed were responsive to Linsitinib treatment, an effect reverted in the absence of both receptors in JIL-2266 and MUC-1. These differences observed among the ACC cell lines underscore the intrinsic heterogeneity of ACC and highlight the complexity of the IGF system in mediating tumor growth. Since it has been recently demonstrated that drug induced cell proliferation inhibition may not correspond to a reduction of cell viability in ACC cells [47], it should be interesting in the future to evaluate also ACC cell lines viability after receptors silencing or inhibition.

At last, in ACC and ACA primary cultures, IGF1R silencing did not affect cell proliferation in either tumour type. Nevertheless, a decrease of cell growth was found in ACC cells silenced for IR, but not in ACA cells. We can hypothesize that IR, expressed as the mitogenic IRA isoform in these tumours, coupled with the overexpression of IGF2 in ACC, but not in ACA, can sustain the growth of ACC cells more than IGF1R. This suggests that novel treatments specifically targeting IRA may be useful in reducing the IGF2-mediated autocrine proliferative loop in IR-expressing ACC. However, given the limited sample size of primary cultures analyzed in this study, due to the rarity of ACC and the limited availability of suitable surgical samples for ACA, future studies with a larger cohort of primary cultures will be required to confirm and extend these findings. Moreover, while we demonstrated a clear involvement of IR in mediating ACC proliferation, further investigation into the underlying biological mechanisms is required. Validation of IR role and its splicing isoforms, along with preclinical studies investigating the therapeutic efficacy of selective IRA inhibitors in in vivo models, are also warranted to provide a more comprehensive understanding of IGF system and potential therapeutic strategies.

## Conclusions

In conclusion, these data demonstrated that IGF1R plasma membrane localisation is more frequent in ACC than in ACA and is associated with a worse tumour behaviour in ACC. Additionally, even if IR is not expressed in all ACC, IR immunopositivity, with IRA as the prevalent isoform, and its plasma membrane localisation are correlated with aggressive features. Furthermore, the experiments performed in ACC cell lines and ACC and ACA primary cultures showed the ability of IR to mediate the IGF2-mitogenic effect in ACC but not in ACA.

Overall, these data suggest that IGF1R and IR expression at the plasma membrane could represent new biomarkers predicting tumour aggressiveness, as well as possible molecular markers useful to patients’ stratification for more individualized IGF1R-IR targeted therapies or for novel pharmacological approaches specifically targeting IRA isoform.

## Electronic supplementary material

Below is the link to the electronic supplementary material.

Supplementary Material 1

Supplementary Material 2

Supplementary Material 3

Supplementary Material 4

## Data Availability

No datasets were generated or analysed during the current study.

## Abbreviations

IGF2: Insulin-like growth factor 2
ACC: Adrenocortical carcinoma
IGF1R: Insulin-like growth factor 1 receptor
IRA: Isoform A of insulin receptor
IR: Insulin receptor
ACA: Adrenocortical adenoma
IRB: Isoform B of insulin receptor
RT-Qpcr: Quantitative real-time PCR
Ki67: Antigen Kiel 67
EDP: Etoposide, doxorubicin, and cisplatin chemotherapy
IGFBP1-6: IGF binding proteins 1–6
IGFBP-rP: IGFBP related proteins
RTK: Tyrosine kinase receptor
INSR: Insulin receptor gene
NAG: Normal adrenal gland
ENSAT: European Network for the Study of Adrenal Tumours
IHC: Immunohistochemistry
FFPE: Formalin-fixed, paraffin embedded samples
OS: Overall survival
PFS: Progression free survival
IRS: Immunoreactivity score
ATCC: American type culture collection
DMEM: Dulbecco’s modified Eagle’s medium
ITS: Insulin-transferrin-selenium
FBS: Fetal bovine serum
BCA: Bicinchoninic acid
SDS: Sodium dodecyl sulfate
NIH: National Institutes of Health
BrdU: 5-bromo-2-deoxyuridine
SD: Standard deviation
IQR: Interquartile range
