# Supplementary figures and images for: Emerging role of IGF1R and IR expression and localisation in adrenocortical carcinomas
Source: Cell Commun Signal. 2025 Mar 4;23:119. doi: 10.1186/s12964-025-02115-0 (PMC11877998; doi:10.1186/s12964-025-02115-0)

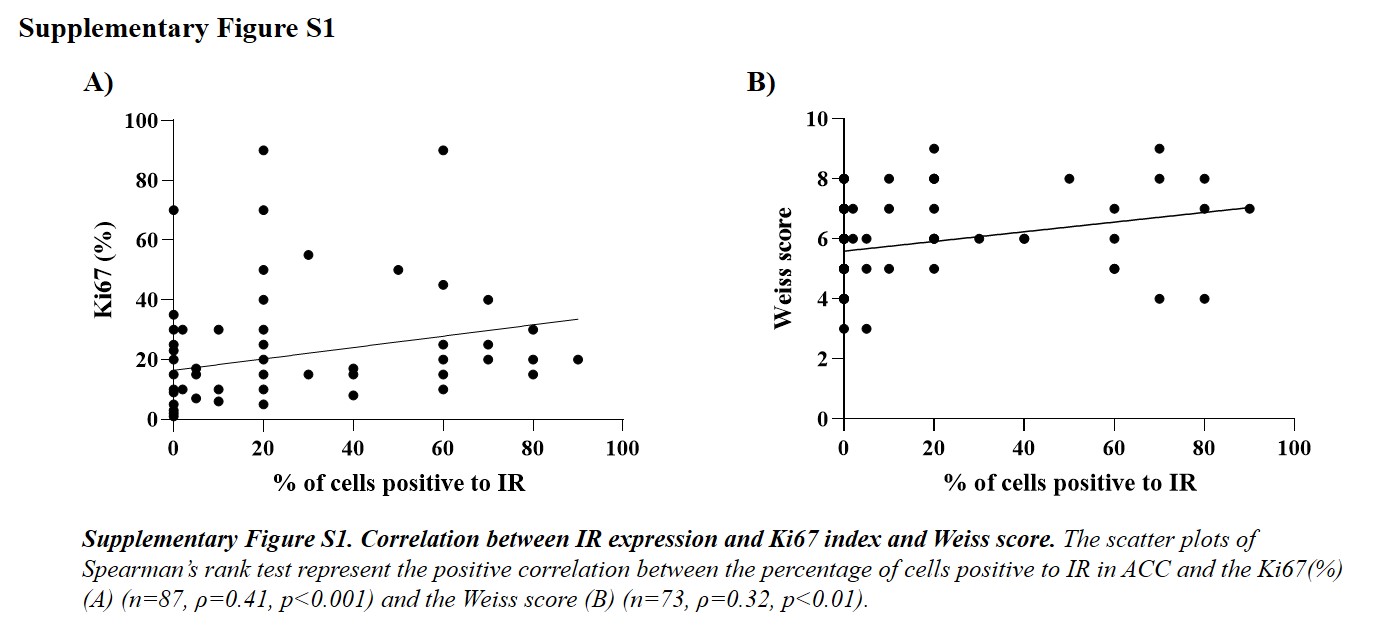

Supplement: Supplementary file 1 — Supplementary Material 1 [file 12964_2025_2115_MOESM1_ESM.jpg]

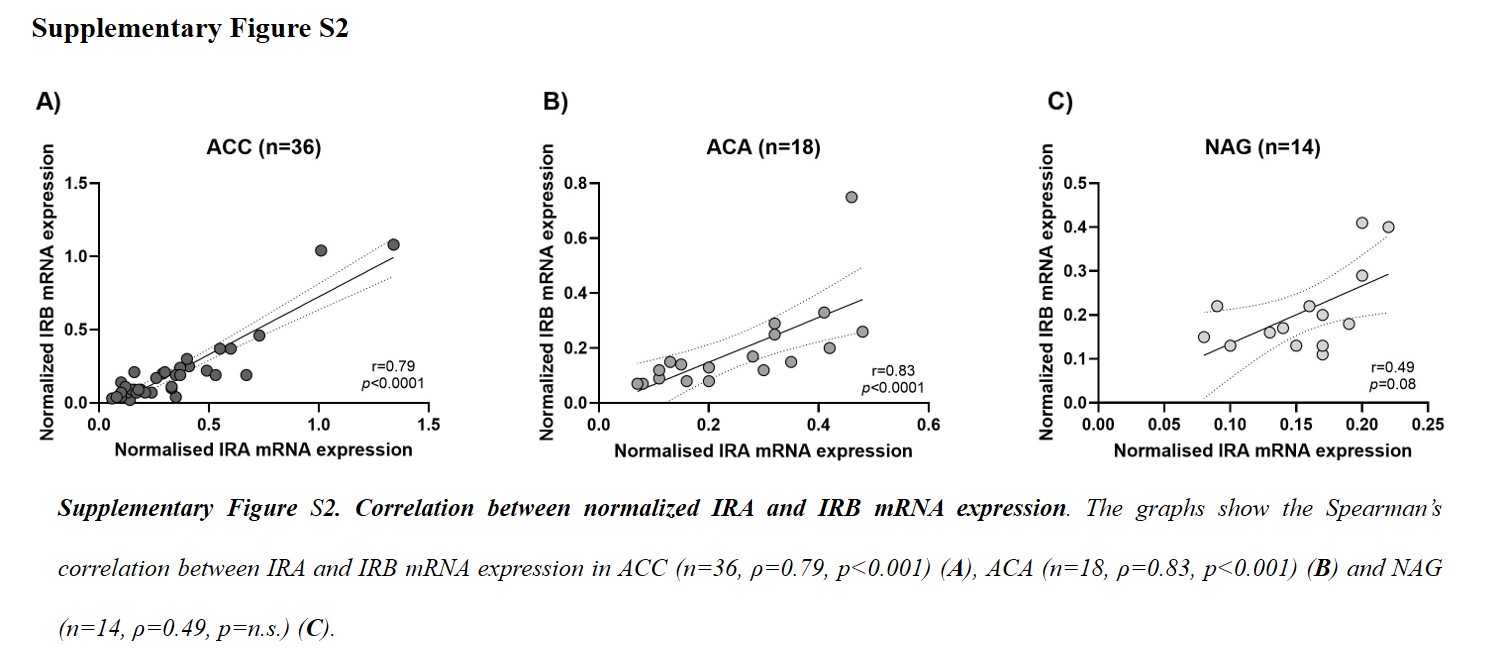

Supplement: Supplementary file 2 — Supplementary Material 2 [file 12964_2025_2115_MOESM2_ESM.jpg]

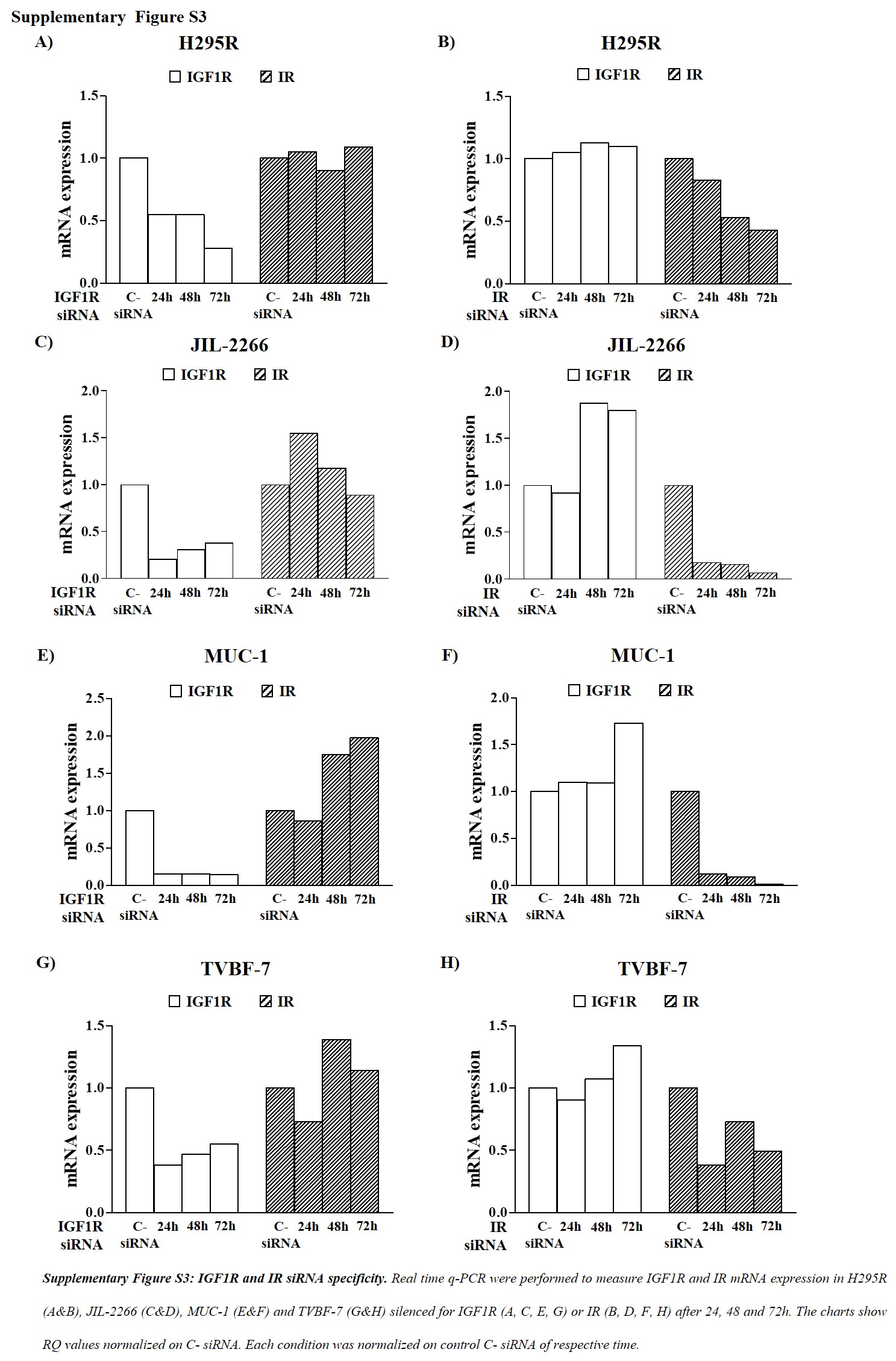

Supplement: Supplementary file 3 — Supplementary Material 3 [file 12964_2025_2115_MOESM3_ESM.jpg]
