# Supplementary material for: Emerging role of IGF1R and IR expression and localisation in adrenocortical carcinomas
Source: Cell Commun Signal. 2025 Mar 4;23:119. doi: 10.1186/s12964-025-02115-0 (PMC11877998; doi:10.1186/s12964-025-02115-0)
Supplement: Supplementary file 4 — Supplementary Material 4 [file 12964_2025_2115_MOESM4_ESM.docx]

**Supplemental Table 1. Primer sequences and probes for the evaluation of IRA and IRB in human tissues.**

| **Primers** | **Sequences** |
| --- | --- |
| IR-F  IR-R | CTGCACAACGTGGTTTTCGT  ACCGTCACATTCCCAACATC |
| IRA-IN  IRB-IN | CAGGCCATCTCGGAAACGC  ACCTCTTCAGGCACTGGTGCC |
| IGF1R-F | ACTCGGACGTCTGGTCCTTC |
| IGF1R-R | CTCGTTGGACAAGCCCTGG |
| IRA-F | TTTTCGTCCCCAGGCCATC |
| IRB-F | CCCCAGAAAAACCTCTTCAGG |
| IRA and IRB-R | GTCACATTCCCAACATCGCC |

*Abbreviation: F, forward; R, reverse; IN, intern.*

**Supplementary Table 2:** Demographic, clinical, and histopathological characteristics of patients with adrenocortical carcinoma (ACC) and adenoma (ACA).

|  | **ACC**  **(n=118)** | **ACA**  **(n=22)** |
| --- | --- | --- |
| **Gender** |  |  |
| Female/Male - n (%) | 75/43 (63.6/36.4) | 14/8 (63.6/36.4) |
| **Age at diagnosis** |  |  |
| ≥50 yrs - n (%) | 58 (49.2) | 15 (78.9) |
| **Type of tissue** |  |  |
| Primary tumor - n (%) | 107 (90.7) | / |
| Local recurrence or distant metastasis – n (%) | 11 (9.3) | / |
| **Hormonal overproduction** |  |  |
| Inactive - n (%) | 35 (34.3) | 0 (0.0) |
| Glucocorticoids +/- others - n (%) | 52 (51.0) | 17 (81.0) |
| Other steroids - n (%) | 15 (14.7) | 4 (19.0) |
| N total | 102 | 21 |
| **ENSAT stage at first diagnosis** |  |  |
| I stage - n (%) | 16 (13.7) | / |
| II stage - n (%) | 46 (39.3) | / |
| III stage - n (%) | 41 (35.0) | / |
| IV stage - n (%) | 14 (12.0) | / |
| N total | 117 | / |
| **Ki67% of the analysed tissues** |  |  |
| 0-9 % - n (%) | 25 (24.3) | / |
| 10-19 % - n (%) | 36 (35.0) | / |
| ≥20 % - n (%) | 42 (40.8) | / |
| N total | 103 | / |
| **Weiss score of the primary tumour** |  |  |
| <6 - n (%) | 36 (41.9) | / |
| ≥6 - n (%) | 50 (58.1) | / |
| N Total | 86 | / |
| **S-GRAS category of the primary tumor** |  |  |
| 0-1 - n (%) | 7 (12.7) | / |
| 2-3 - n (%) | 23 (41.8) | / |
| 4-5 - n (%) | 16 (29.1) | / |
| 6-9 - n (%) | 9 (16.4) | / |
| N total | 55 | / |
| **Follow-up of the primary tumour (months)**  median (IQR) | 39.5 (54.7) | 39.5 (21.5) |
| N total | 100 | 18 |

*ACC, adrenocortical carcinoma; ACA, adrenocortical adenoma; IQR, range interquartile.*

**Supplementary Table 3. The individual histological Weiss parameters in ACC with or without IGF1R membrane localisation.**

| **Histological Weiss criteria** | **IGF1R plasma membrane localisation**  **(%)** | **IGF1R no plasma membrane localisation**  **(%)** | **p value** | | |
| --- | --- | --- | --- | --- | --- |
| **Nuclear grade** |  |  |  | | |
| High | 88.9 | 69.2 | 0.36 | | |
| Low | 11.1 | 30.8 |  |  |  |
| **Mitoses** |  |  |  | | |
| ≤5 per 50 high-power fields | 0.0 | 46.2 | ***0.046*** | | |
| >5 per 50 high-power fields | 100.0 | 53.8 |  |  |  |
| **Atypical mitoses** |  |  |  | | |
| No | 44.4 | 92.3 | ***0.02*** | | |
| Yes | 55.6 | 7.7 |  |  |  |
| **Clear cells** |  |  |  | | |
| >25% | 11.1 | 0.0 | 0.41 | | |
| ≤25% | 88.9 | 100.0 |  |  |  |
| **Diffuse architecture** |  |  |  | | |
| ≤33% surface | 0.0 | 0.0 | / | | |
| >33% surface | 100.0 | 100.0 |  |  |  |
| **Confluent necrosis** |  |  |  | | |
| No | 22.2 | 69.2 | 0.08 | | |
| Yes | 77.8 | 30.8 |  |  |  |
| **Venous invasion** |  |  |  | | |
| No | 22.2 | 84.6 | ***0.007*** | | |
| Yes | 77.8 | 15.4 |  |  |  |
| **Sinusoidal invasion** |  |  |  |  |  |
| No | 33.3 | 53.8 | 0.41 | | |
| Yes | 66.7 | 46.2 |  |  |  |
| **Capsular infiltration** |  |  |  | | |
| No | 11.1 | 53.8 | 0.07 | | |
| Yes | 88.9 | 46.2 |  |  |  |

*p values obtained from Fisher’s exact test*

| **Ki67≥10** | **Univariate** | | | | **Multivariate** | | | |
| --- | --- | --- | --- | --- | --- | --- | --- | --- |
| **Variables** | *n* | *OR* | *95%CI* | *p* | *n* | *OR* | *95%CI* | *p* |
| **IGF1R**  **expression** | 99 |  |  |  | 86 |  |  |  |
| High | 20 | 0.74 | [0.2-2.2] | 0.58 | 20 | 0.3 | [0.1-1.3] | 0.1 |
| Low | 79 |  |  |  | 66 |  |  |  |
| **IGF1R plasma membrane localization** | 86 |  |  |  | 86 |  |  |  |
| Yes | 38 | 10.8 | [2.3-50.3] | ***0.002*** | 38 | 14.5 | [2.8-74.9] | ***0.001*** |
| Not | 48 |  |  |  | 48 |  |  |  |

**Supplementary Table 4. Univariate and multivariate logistic regression analysis for risk to have a Ki67≥10 in ACC cohort.**

OR, Odds ratio; CI, confidence interval; *n,* number of evaluated patients.

**Supplementary Table 5. Univariate logistic regression analysis for risk to have a Ki67≥10 and Weiss score≥6 in ACC cohort.**

| **Ki67≥10** | **Univariate** | | | |
| --- | --- | --- | --- | --- |
| **Variables** | *n* | *OR* | *95%CI* | *p* |
| **IR expression** | 87 |  |  |  |
| Yes | 46 | 4.2 | [1.3-12.8] | ***0.01*** |
| No | 41 |  |  |  |
| **IR plasma membrane localisation** | 41 |  |  |  |
| Yes | 30 | 16.6 | [1.5-172] | ***0.02*** |
| No | 11 |  |  |  |
| **Weiss score≥6** | **Univariate** | | | |
| **Variables** | *n* | *OR* | *95%CI* | *p* |
| **IR expression** | 73 |  |  |  |
| Yes | 41 | 3.5 | [1.3-9.5] | ***0.02*** |
| No | 32 |  |  |  |
| **IR plasma membrane localisation** | 32 |  |  |  |
| Yes | 25 | 7.0 | [1.1-44.06] | ***0.04*** |
| No | 7 |  |  |  |

*OR, odds ratio; CI, confidence interval; n, number of evaluated patients.*

**Supplementary Table 6. Clinical and pathological characteristics of patients evaluated by qRT-PCR.** The total number of patients in each group is considered if not otherwise specify.

|  | **ACC**  **(n=36)** | **ACA**  **(n=18)** | **NAG**  **(n=14)** |
| --- | --- | --- | --- |
| **Gender - n (%)**  Female/Male | 22/14 (61.1/38.9) | 14/8 (63.6/36.4) | 8/6 (57.2/42.8) |
| **Age ≥50 years old** **at diagnosis - n (%)** | 18 (50.0) | 15 (78.9) | 6 (33.3%) |
| **Type of tissue- n (%)**  Primary tumour  Local recurrence or distant metastasis | 30 (8.3)  6 (16.7) | /  / | /  / |
| **Hormone excess - n (%)**  Inactive  Glucocorticoids +/- others  Other steroids  N available | 6 (18.7)  23 (71.9)  3 (9.4)  32 | 5 (27.8)  10 (55.5)  3 (16.7)  18 | /  /  /  / |
| **ENSAT stage at first diagnosis - n (%)**  I  II  III  IV | 2 (5.5)  15 (41.7)  13 (36.1)  6 (16.7) | /  /  /  / | /  /  /  / |
| **Ki67% of the analysed tissues - median (IQR)** | 17 (20) | / | / |
| **Weiss score of the primary tumor – mean ± SD**  N available | 7 (2)  31 | / | / |
| **S-GRAS category of the primary tumor - n (%)**  0-1  2-3  4-5  6-9  N available | 4 (12.5)  12 (37.5)  10 (31.2)  6 (18.8)  32 | /  /  /  /  / |  |

*N, number; ACA, adrenocortical adenoma; ACC, adrenocortical carcinoma; N, number; NAG, normal adrenal gland; IQR, range interquartile.*
